# The effect of the duration of intravenous zolendronate medication on the success of non-surgical endodontic therapy: a retrospective study

**DOI:** 10.1186/s12903-016-0163-6

**Published:** 2016-02-01

**Authors:** Ömür Dereci, Ekim Onur Orhan, Özgür Irmak, Sinan Ay

**Affiliations:** Faculty of Dentistry, Department of Oral and Maxillofacial Surgery, Eskişehir Osmangazi University, Meşelik Campus, 26480 Eskişehir, Turkey; Faculty of Dentistry, Department of Endodontics, Eskişehir Osmangazi University, Eskişehir, Turkey; Faculty of Dentistry, Department of Restorative Dentistry, Eskişehir Osmangazi University, Eskişehir, Turkey

**Keywords:** Bisphosphonate, Endodontic success, Periapical healing, Zolendronate

## Abstract

**Background:**

Aim of this study is to compare the clinical and radiographic success of non-surgical endodontic therapy in patients receiving intravenous zolendronate less than 1 year and more than 1 year.

**Methods:**

The clinical and radiographic follow-up data of 24 patients who were receiving IV zolendronate with 37 teeth were retrieved from the archives to evaluate clinical and radiographic healing at the end of 12 months after non-surgical endodontic therapy. The clinical and radiographic scores of teeth treated with non-surgical endodontic therapy were analyzed.

**Results:**

The amount of non-healed and incomplete healed teeth in patients receiving zolendronate more than 1 year were more than the amount of teeth of non-healed and incomplete healed in patients receiving bisphosphonates less than 1 year (*p* < 0.05).

**Conclusions:**

There was a strong relationship between the duration of the bisphosphonate medication and endodontic success.

## Background

Bisphosphonate-related osteonecrosis of the jaws (BRONJ) is seen occasionally in bisphosphonate receiving patients for several medical conditions such as osteoporosis, Paget’s disease or metastatic cancer [1]. BRONJ was first described by Marx who observed an association between long-term bisphosphonate medication and exposed necrotic bone of the jaws [2]. The term BRONJ was later changed to MRONJ (medication-related osteonecrosis of the jaws) to accommodate the growing number of gnathic osteonecrosis cases associated with other antiresorptive (denasumab) and antiangiogenic drugs [3]. In position paper published in 2009 [4], it was stated that MRONJ diagnosis is made with the occurrence and persistence of necrotic bone on maxilla and mandible more than 8 weeks in patients who have been using bisphosphonates and have not received radiation on oral and maxillofacial region. However, the diagnosis guidelines have been changed in a latest position paper and 3 criteria were proposed for the correct diagnosis of MRONJ [3]. Patients may be considered to have MRONJ if all of the following characteristics are present:

1. Current or previous treatment with antiresorptive and/or antiangiogenic agents,
2. Exposed bone that can be probed through an intraoral or extraoral sinus tract in the maxillofacial region that has persisted for more than 8 weeks.
3. No history of radiation therapy to the jaws or obvious metastatic disease to the jaws.

Surgical invasive procedures, ill-fitting partial/total dentures or trauma are considered to be responsible for occurrence of MRONJ [5–7]. However, the most common trigger factor for the development of MRONJ is dental extraction [6, 7]. Procedures that involve direct osseous injury should be avoided in patients using bisphosphonates. As dental extraction causes direct trauma to the bone, dental extractions are contraindicated if the patient is under antiresorptive drug medication [3]. If not-restorable or damaged teeth are present in bisphosphonate receiving patients; removal of the crown of the damaged tooth followed by endodontic treatment of the remaining roots is recommended [4]. Root canal treatment (RCT) in bisphosphonate receiving patients is a safe procedure and the prevalence of MRONJ may be reduced [4]. There are several studies that encourage RCT in patients receiving bisphosphonates [8–10]. However, success of RCT among patients receiving bisphosphonates in different periods has not been evaluated yet.

Aim of this study is to compare the success of non-surgical RCT in patients receiving bisphosphonate medication less than 1 year and more than 1 year.

## Methods

### Study design

Thirty-two zolendronate (ZOMETA®, Novartis Pharmaceuticals Co., Basel, Switzerland) medicated patients who were referred to department of endodontics from department of oral and maxillofacial surgery for non-surgical RCT or re-treatment of teeth with infected roots between 2008 and 2012 were selected for this retrospective study. The current study looked retrospectively at outcomes for a cohort of patients treated and does not report experimental or new protocols. All data analysed were collected as part of routine diagnosis and treatment. Therefore, ethical approval was not needed. Inclusion criteria of the cases were as follows;

1. Single or multi root teeth treated with multiple visit non-surgical RCT, (The root canal treatment of all patients were finished in 2012).
2. Twelve-months follow-up period without any delay or drop,
3. Fully achievable medical records and radiographs,
4. Patients who were ‘at risk’ stage according to MRONJ staging category [3].

Twenty-four patients were included in the study according to the criteria. All clinical and follow-up data of 24 patients were retrieved from the archives. All patients had symptomatic or asymptomatic apical periodontitis and were receiving 4 mg/3 weeks intravenous zolendronate at the time of the RCT.

### Endodontic therapy

Informed consent forms were read and signed by all patients prior to the RCT. All RCTs were performed by a single endodontist with 10 years of clinical experience using Reciproc® system (VDW GmBH, Germany) as previously described [11]. Using the instrument in a brushing motion facilitated removal of infected debris from root canals. Same protocol was also used removing gutta-percha remnants in retreatment cases with any additional chemicals for dissolving gutta-percha cones. Working length was determined using an electronic apex locator (Raypex 5, VDW GmBH, Germany) with reading between the yellow zone and apex marks. Root canals were frequently irrigated with 2.5 % sodium hypochlorite during canal preparation procedures. Smear layer removal protocol of sequential use of 5 mL of 2.5 % sodium hypochlorite, 5 mL 17 % EDTA and 2 mL 2 % chlorhexidine for 1 min with intermediate flushing between each irrigation solution. Root canals were filled with calcium hydroxide (Calcipast, Cerkamed Medical Co., Stalowa Wola, Poland) as an interim dressing for a period of 14 days. Cavities were then sealed with cement to prevent bacterial leakage. In the second visit, calcium hydroxide was removed by 40 % citric acid (Cerkamed Medical Co.), root canals were dried with paper points (Reciproc® paper points, VDW GmBH) and canals were obturated with Reciproc® (VDW GmBH) single gutta-percha point and root canal sealer (AHPlus® De Trey, Konstanz, Germany) followed by sealing of cavities with temporary cement. After 24 h, cement was removed and cavity was incrementally restored by a single operator with nano-hybrid resin composite (Filtek Z550, Shade A1, 3 M Espe, USA) using two-step self-etching adhesive system (Clearfil SE Bond, Kuraray Inc, Japan) according to manufacturers’ instructions.

Clinical success of non-surgical RCTs was evaluated according to the criteria as follows [9];

1. Total Healing: asymptomatic, functional tooth with minimal or no apical lesion.
2. Functional Healing: asymptomatic, functional tooth with apical lesion.
3. Non-Healing: symptomatic, non-functional tooth with or without apical lesion.

### X-ray evaluation

Standardized digital radiographs (RadioVisioGraphy, Trophy Radiologie, Marne-La Vallée, France) of preoperative and corresponding recall sessions were sequentially examined by three independent clinicians (O.O.E., D.O. and I.O.) using an image-analysis software (Adobe Photoshop CC, Adobe Systems Inc., San Jose, Calif., USA) at a magnification ratio of 1:1 on a 22” plug and play LED monitor attached to a computer equipped with NVIDIA GeForce GTX 980 graphic card giving 2048 × 1536 pixels of resolution in a 4000 K day-lit room. Inter-observer agreement was assessed by using Cohen’s kappa test [12]. The examiners were calibrated before the evaluation. Training was conducted on images of periapical healing of 40 cases not present in the current study. A kappa value of 0.83 was calculated following this calibration exercise. In case of disagreement, images were re-evaluated until consensus was achieved. Three examiners re-rated the same set of radiographs for intra-observer reproducibility after 2 week interval. The intra-observer kappa values were 0.93, 0.89, and 0.87 respectively.

**Fig. 1:**
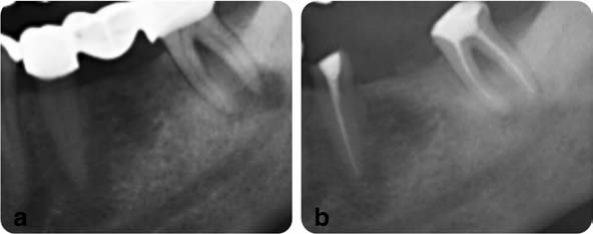
A representative x-ray image of complete healing, **a** before RCT, **b** a-year follow-up

X-ray scores were set as follows;

1. Complete healing (CH): absence or no development of apical lesion or only small changes in bone structure on recall radiograph (Fig. 1)

.
2. Incomplete healing (IH): obvious reduction of apical lesion but repair process still is incomplete on recall radiograph (Fig. 2).

**Fig. 2:**
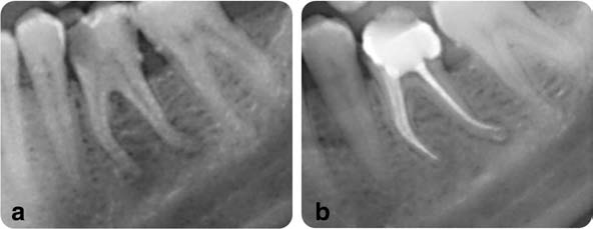
A representative x-ray image of incomplete healing, **a** before RCT, **b** a-year follow-up
3. No healing (F): apical lesion was enlarged/unchanged, or a new apical lesion observed on recall radiograph (Fig. 3).

**Fig. 3:**
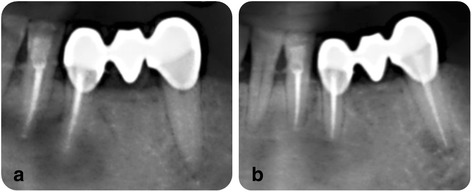
A representative x-ray image of non-healing, **a** before RCT, **b** a-year follow-up

### Statistical analysis

Statistical analysis was performed with SPSS 20.0 packet program. Chi square test was used to define the difference between clinical and radiographic success levels of non-surgical RCTs in patients receiving biphosphonates less than 1 year and more than 1 year (*p* < 0.05).

## Results

Clinical data of all patients is shown in Table 1. The mean age of all patients were 60.2 (±10.9), ten patients were male and 14 patients were female. Nineteen patients were diagnosed with metastatic bone disease and five of them were diagnosed with malignancy related hypercalcemia at the time of the endodontic therapy. The total amount of teeth that were treated with non-surgical endodontic therapy was 37. Sixteen patients (76.7 %) were receiving zolendronate more than 1 year and eight patients (33.3 %) were receiving zolendronate less than 1 year. 8.1 % of 37 teeth were treated with non-surgical re-treatment. All patients were still receiving zolendronate at recall examinations. The number of the teeth scored as non-healing and functional healing in clinical examination of patients receiving zolendronate more than 1 year was higher than patients receiving zolendronate less than 1 year in the 12 month recall examination. There was strong evidence of a relationship between the duration of zolendronate medication and endodontic success (*p* < 0.05).

**Table 1:** Clinical data of all study patients

Patient		# Tooth (FDI)	Age	Gender	Duration of IV zolendronat medication		Recall Time	Treatment type	Clinical Healing Score			X-ray score		
					Less than a year	More than a year			Complete healing	Functional healing	Non-Healing	CH	IH	F
1.	1	45	57	F		+	12	NS-RCT		+			+	
2.	1	44	57	F		+	12	NS-RCT		+			+	
3.	2	34	46	F	+		12	NS-RCT	+			+		
4.	2	37	46	F	+		12	NS-RCT	+				+	
5.	2	42	46	F	+		12	NS-RCT	+			+		
6.	3	45	61	M		+	12	NS-RCT		+			+	
7.	4	33	73	M		+	12	NS-RCT			+		+	
8.	5	36	47	F		+	12	NS-RCT		+			+	
9.	6	43	41	M	+		12	Re-T	+			+		
10.	6	46	41	M	+		12	Re-T	+				+	
11.	7	47	62	F		+	12	NS-RCT			+			+
12.	8	35	56	F	+		12	NS-RCT	+			+		
13.	8	37	56	F	+		12	NS-RCT		+			+	
14.	9	21	71	M		+	12	NS-RCT		+			+	
15.	9	22	71	M		+	12	NS-RCT		+			+	
16.	10	33	49	M		+	12	NS-RCT		+			+	
17.	10	34	49	M		+	12	NS-RCT		+			+	
18.	11	31	62	F	+		12	NS-RCT	+				+	
19.	11	41	62	F		+	12	NS-RCT		+			+	
20.	12	15	81	F		+	12	NS-RCT	+				+	
21.	13	22	77	F		+	12	NS-RCT		+			+	
22.	13	23	77	F		+	12	NS-RCT		+			+	
23.	14	47	58	M		+	12	NS-RCT		+			+	
24.	15	44	55	F	+		12	NS-RCT	+			+		
25.	15	47	55	F		+	12	NS-RCT		+			+	
26.	16	33	61	F		+	12	NS-RCT		+			+	
27.	16	37	61	F		+	12	NS-RCT		+			+	
28.	17	12	79	M	+		12	Re-T			+			+
29.	18	17	51	F		+	12	NS-RCT		+			+	
30.	18	25	51	F		+	12	NS-RCT		+			+	
31.	19	44	64	F	+		12	NS-RCT	+				+	
32.	20	42	67	M		+	12	NS-RCT			+			+
33.	21	31	59	M		+	12	NS-RCT		+			+	
34.	22	24	46	M		+	12	NS-RCT		+			+	
35.	23	17	54	F	+		12	NS-RCT		+			+	
36.	23	14	54	F		+	13	NS-RCT		+			+	
37.	24	22	68	F		+	12	NS-RCT		+			+	

*Re-T* Non-surgical root canal retreatment, *NS-RCT* Non-surgical root canal treatment, *CH* Complete healing, *IH* Incomplete healing, *F* No healing

Similar to the clinical healing scoring, the amount of non-healed and incomplete healed teeth in patients receiving zolendronate more than 1 year were more than non-healed and incomplete healed teeth in patients receiving zolendronate less than 1 year. There was a strong evidence of a relationship between radiographic success and duration of zolendronate medicatio006E (*p* < 0.05).

## Discussion

Bisphosphonates are pyrophosphate analogues with antiresorptive properties. They can be classified into two categories as nitrogen containing and non-nitrogen containing according to structural differences in molecular chains. Nitrogen containing bisphosphonates such as zolendronate and pamidronate are potent molecules and inhibit osteoclast activity more than non-nitrogen containing bisphosphonates [13]. There is a positive correlation between the duration and accumulative dosage of bisphosphonates [10].

The pathophysiology of MRONJ has not been fully elucidated yet [2, 14]. Several hypotheses were postulated in an attempt to explain the pathologic mechanism of the bone necrosis of the jaws. The unique localization of bone necrosis in MRONJ may be attributed to major factors such as altered bone remodeling, hampered bone resorption and constant micro trauma and auxiliary factors such as suppression of innate and acquired immunity, soft tissue bisphosphonate toxicity, angiogenesis inhibition and inflammation [5, 15–18]. Osteoclast differentiation and function play a crucial role in bone healing and remodeling in all skeletal bones [19]. Jaw bones have increased remodeling rate compared to any other skeletal bones, therefore, bone remodeling in jaws is more easily inhibited in bisphosphonate medicated patients, revealing healing disturbances after dental procedures such as tooth extraction and root-canal treatment [20]. Staging and management of MRONJ is clearly defined in the position paper in 2004 [4]. Besides an appropriate management of the disease, prevention of MRONJ with a multi-disciplinary approach is an important issue in pre-administration phase or ‘at-risk’ phase in patients receiving bisphosphonates. Several studies reported that appropriate dental screening and necessary dental treatment before bisphosphonate medication reduced the risk of MRONJ [21, 22]. In patients with ongoing bisphosphonate medication, especially oncology patients receiving monthly intravenous bisphosphonate therapy, invasive dental procedures that may possess a risk of bone damage such as tooth extraction or dentoalveolar surgery should be avoided if possible [4]. If necrosis develops in bisphosphonate medicated cancer patients, oncologist may consider to suspend bisphosphonate therapy until healing of osteonecrosis occurs [4]. Estilo et al. [23] reported that the interruption or decrease in bisphosphonate therapy did not seem to alter the course of MRONJ.

Endodontic therapy, as a conservative method, is gold standard for the treatment of symptomatic teeth in patients receiving bisphosphonate medication. Although endodontic treatment is considered safe in bisphosphonate receiving patients, it may induce MRONJ as well [4]. Moinzadeh et al. [10] suggested that non-surgical endodontic treatments in patients medicated with bisphosphonates should be done with extreme caution due to the risk of MRONJ. In spite of a possibility of MRONJ development as a cause of endodontic therapy in patients receiving bisphosphonates, it is still commonly accepted that non-surgical endodontic treatment is much more favorable than dental extractions in patients receiving intravenous bisphosphonates in long periods [10].

There are a limited number of studies that investigate the benefits of endodontic therapy in patients receiving bisphosphonates. Hsiao et al. [9] reported that there was no significant difference in peri-radicular healing in patients receiving and not receiving oral bisphosphonates. In contrary, there was strong evidence of a relationship between the duration of bisphosphonate medication and the reduction of the periapical pathology in the current study (*p* < 0.05). Hsiao et al. [9] included patients receiving oral bisphosphonates in their study. This may be the cause of the different results of the study of Hsiao et al. and the current study in which only patients with IV zolendronate medication were included. The comparison of the peri-radicular and clinical healing in patients receiving bisphosphonates for more than 1 year and less than 1 year was made first time in the current study to our knowledge.

The duration of bisphosphonate or antiresorptive medication is a continuous risk factor for the development of MRONJ regardless of the indication for the medical therapy. The incidence of MRONJ development among cancer patients receiving denosumab and zolendronate was respectively 0.5–0.8 % at 1 year and 1.0–1.8 % at 2 years, 1.3–1.8 at 3 years [24]. Difference between incidences of MRONJ occurrence of 1 year and 2 years continuous antiresorptive medication is prominent. Therefore, 1 year is a turning point after which the possibility of developing MRONJ prominently increases. However, the risk of MRONJ development among patients who have received bisphosphonate medication for endodontic therapy is unknown [3]. For that particular reason, in the current study, 1 year time was taken as turning point to evaluate the success of RCT among zolendronate receiving patients.

## Conclusion

In the current study, it can be concluded that non-surgical endodontic therapy is more successful in patients receiving zolendronate less than 1 year. Our study is the first study that compares the success of non-surgical RCT with the duration of bisphosphonate medication in ‘at risk’ group of patients receiving bisphosphonates to our knowledge. One disadvantage of the current study is that it is a retrospective study with limited sample size. Prospective studies with larger sample sizes should be conducted in order to achieve more favorable results.

## Abbreviations

BRONJ: Bisphosphonate-related osteonecrosis of the jaws
MRONJ: Medication-related osteonecrosis of the jaws
RCT: Root canal treatment
